# Local efficacy and survival outcome of salvage endoscopic therapy for local recurrent lesions after definitive chemoradiotherapy for esophageal cancer

**DOI:** 10.1186/s13014-016-0604-z

**Published:** 2016-02-27

**Authors:** Ken Hatogai, Tomonori Yano, Takashi Kojima, Masakatsu Onozawa, Satoshi Fujii, Hiroyuki Daiko, Yusuke Yoda, Takuya Hombu, Toshihiko Doi, Kazuhiro Kaneko, Atsushi Ohtsu

**Affiliations:** Department of Gastroenterology, Endoscopy Division, National Cancer Center Hospital East, 6-5-1, Kashiwanoha, Kashiwa, Chiba 277-8577 Japan; Department of Gastroenterology, Gastrointestinal Oncology division, National Cancer Center Hospital East, Kashiwa, Chiba Japan; Department of Medical Oncology, Graduate School of Medicine, Chiba University, Chuo-ku, Chiba Japan; Department of Radiation Oncology, National Cancer Center Hospital East, Kashiwa, Chiba Japan; Division of Pathology, Exploratory Oncology Research & Clinical Trial Center, National Cancer Center, Kashiwa, Chiba Japan; Department of Esophageal Surgery, National Cancer Center Hospital East, Kashiwa, Chiba Japan; Exploratory Oncology Research & Clinical Trial Center, National Cancer Center, Kashiwa, Chiba Japan

**Keywords:** Photodynamic therapy, Endoscopic mucosal resection, Chemoradiotherapy, Salvage treatment, Esophageal cancer, Local recurrence

## Abstract

**Background:**

Salvage endoscopic therapy (SET), such as endoscopic mucosal resection (EMR) and photodynamic therapy (PDT), is a less-invasive treatment for local failure at the primary site after chemoradiotherapy (CRT) for esophageal squamous cell carcinoma (ESCC). We conducted this retrospective study to clarify the risk factors for local recurrence along with the long term results after SET for recurrent lesions after definitive CRT for ESCC.

**Methods:**

We enrolled 77 consecutive patients who underwent EMR or PDT for local recurrence without any metastasis after definitive CRT at our institution. We evaluated the local efficacy, local recurrence-free survival (LRFS), and overall survival (OS), and investigated the risk factors associated with survival outcome using a multivariate analysis.

**Results:**

The complete resection rate of EMR was 84.6 % (33/39), and the complete response rate for PDT was 65.8 % (25/38). Twenty-two patients (28.6 %) exhibited local recurrence without metastasis. Thirty-four patients (44.2 %) were alive at 5 years after undergoing only initial SET or with repeated SET. The 5-year LRFS rate was 59.6 %, and the presence of lesions occupying an esophageal circumference of 1/4 or larger was the only significant risk factor (HR: 3.10, 95 % CI: 1.35–7.15, *P* = 0.008). The 5-year OS rate was 48.4 %, and an advanced T factor before CRT was marginally associated with a poor OS (HR: 1.96, 95 % CI: 0.98–3.92, *P* = 0.055).

**Conclusions:**

SET enabled a preferable local control and survival outcome for patients with local recurrence after definitive CRT for ESCC. Careful endoscopic follow-up is needed for patients with a large lesion before SET and those with an advanced T factor before CRT.

**Electronic supplementary material:**

The online version of this article (doi:10.1186/s13014-016-0604-z) contains supplementary material, which is available to authorized users.

## Introduction

Concurrent chemoradiotherapy (CRT) is a definitive treatment option for locally advanced esophageal squamous cell carcinoma (ESCC) [1, 2]. Even if a complete response is achieved, locoregional recurrence often occurs and is a major obstacle to achieving a cure [3–5]. According to the current National Comprehensive Cancer Network guideline, an esophagectomy can be proposed for patients with locoregional recurrence if the lesion is resectable and the patient is medically operable, while chemotherapy or palliative care should be proposed if the lesion is not resectable or if the patient is not medically operable [6]. However, an esophagectomy following definitive CRT is associated with a higher incidence of postoperative adverse events and mortality, compared with both primary surgery and planned surgery after neoadjuvant CRT [7–10]. Therefore, no established standard therapy exists for relatively shallow recurrent lesions limited to the esophagus. Reportedly, patients who have achieved a complete response with CRT are very unlikely to experience a recurrence at locoregional lymph nodes [11]. In patients with local recurrence at the primary site and without any recurrence in the lymph nodes, only treatment for the local recurrent lesion is capable of achieving a cure. Given the importance of local control at the primary site and the possible adverse events related to salvage surgery, salvage treatment at the primary site using endoscopy could be a curative and less invasive option in select patients.

We have performed two kinds of salvage endoscopic therapy (SET), endoscopic mucosal resection (EMR) and photodynamic therapy (PDT), as optional treatments for local failure (such as local residual lesions after CRT and local recurrent lesions after the achievement of a complete response after CRT) and have reported favorable efficacy and safety results for each treatment [12, 13]. Furthermore, local recurrent lesions were thought to be good candidates for SET [13]. While EMR is relatively concise and is a widely used procedure, it is only indicated for lesions within the shallow submucosal layer. In contrast, PDT can be used for lesions that have invaded even the muscularis propria layer; however, this technique is associated with phototoxicity requiring a long period of avoiding sunlight. The significance of SET is its ability to cure the local recurrent lesion while allowing organ preservation. However, patients undergoing SET often experience local recurrence after the initial SET, and some of these patients can be rescued by an additional SET. If the risk factors for local recurrence are clarified, intensive endoscopic observations could be performed for patients with a high risk. To date, no study has integrated these treatment outcomes in a comprehensive assessment. The aim of the present study was to clarify the risk factors for local recurrence after SET along with the long-term results of SET after definitive CRT for ESCC.

## Patients and methods

### Patients

We enrolled consecutive patients with a local recurrent lesion who underwent SET at the National Cancer Center Hospital East in this retrospective study according to the following selection criteria among patients who underwent definitive CRT for ESCC at the National Cancer Center Hospital East between 1998 and 2008 and patients referred from other hospitals for salvage treatment after definitive CRT during the same period: 1) received definitive CRT prior to SET, which consisted of external beam irradiation of 50 Gy or more along with chemotherapy; and 2) absence of any lymph node or distant metastasis as determined using computed tomography (CT) before SET. The study protocol was approved by the institutional review board of the National Cancer Center Hospital East (2012–275). The study was carried out according to the ethical principles of the Declaration of Helsinki and the Epidemiological Study Guideline of Ministry of Health, Labour and Welfare in Japan. For the deceased patients and their relatives, we disclosed the study design on the website of the National Cancer Center and gave them the opportunity to refuse participation in this retrospective study.

### Evaluation before CRT and SET

Clinical staging before CRT was determined by endoscopy, endoscopic ultrasound (EUS), and contrast enhanced CT according to the TNM Classification of the International Union Against Cancer [14]. Positron emission tomography was an option before CRT and before SET. All the patients underwent endoscopy and contrast CT after the completion of CRT to evaluate the response. A complete response after CRT was determined when an endoscopic examination showed the disappearance of the primary tumor and the absence of cancer cells in biopsy specimens, in addition to the achievement of a complete response according to the Response Evaluation Criteria in Solid Tumors on a CT scan [15].

Local recurrent lesions were defined as follows: 1) recurrence after having once achieved a complete response after CRT, in the area where the primary tumor had existed prior to CRT; and 2) biopsy findings were positive for cancer cells from the recurrent lesions. Before SET, all the patients were evaluated and staged using EUS (EU-M2000; Olympus Co. Ltd., Tokyo, Japan) with a high-frequency (20 MHz) ultrasound probe. Lesions were classified as either uT1 (limited to the submucosal layer) or uT2 (limited to the muscularis propria layer) [16]. The lesion circumference was classified as follows based on Lugol’s chromoendoscopy and EUS findings: <1/4; ≥1/4 and <1/2; ≥1/2 and <3/4; ≥3/4.

### Indications for salvage endoscopic therapy and criteria for treatment choice

The indications for SET were as follows: 1) local recurrence limited to within uT2, as determined using EUS and CT; 2) the absence of lymph node or distant organ metastasis when a local recurrence was detected; and 3) patients’ refusal to undergo salvage surgery or a physical condition that would have made surgery intolerable. Since PDT requires an approximately 4-week period of no exposure to sunlight after the administration of a photosensitizer, EMR was typically chosen for SET for local recurrence because of convenience if the lesion fulfilled the following criteria: 1) absence of ulceration on the lesion, and 2) limited to the shallow submucosal layer as determined using EUS. If ulceration or fibrosis was present in the lesions or the depth of the lesion was suspected to be the deep submucosal layer or deeper, PDT was indicated for SET.

### Procedures

The technique used for salvage EMR consisted of the strip biopsy method [17]. Briefly, after the submucosal injection of saline solution, EMR was performed using a dual channel endoscope (2 T240; Olympus, Tokyo, Japan). The resected specimens were pathologically evaluated by experienced pathologists. A complete resection was determined based on both clinical and pathological evaluations. For the clinical evaluation, the edge of the post-EMR ulcer was carefully assessed using Lugol’s chromoendoscopy just after EMR; if the unstained area had been completely removed, a clinically complete resection was defined. For the pathological evaluation, a complete resection after EMR with en bloc resection was defined when cancer cells were not observed at the horizontal and vertical resection margins of the specimens when examined microscopically. For EMR with a piecemeal resection, a complete resection was defined when cancer cells were not observed at the supposed resection margin after the resected pieces had been reconstructed. In this study, a complete resection was required to have fulfilled both the clinical and pathological definitions.

PDT was performed using an excimer dye laser (EDL-1; Hamamatsu Photonics, Hamamatsu, Japan), as previously reported [13]. Briefly, the procedure was initiated with the intravenous administration of a photosensitizer, porfimer sodium (Pfizer Japan Inc., Tokyo, Japan), followed by 630-nm-wavelength excimer dye laser irradiation 48 h later. We routinely performed an endoscopy on the following day, and if an obvious residual tumor was found, an additional laser irradiation was performed. A complete response was regarded as the disappearance of the tumor lesion and ulceration after PDT, as verified by endoscopic examination, and the absence of cancer cells in the biopsy specimens. In all the cases, the EMR and PDT procedures were performed by 3 doctors certified by the Japan Gastroenterological Endoscopy Society (TY, TK, and YY). Representative images of patients who underwent EMR and PDT are presented in Additional file 1: Figure S1 and Additional file 2: Figure S2, respectively.

A follow-up examination consisting of endoscopy and CT was performed to search for local recurrence and lymph node or distant organ metastases every 3 months for the first 2 years after SET and every 6 months thereafter.

### Statistical analysis

Differences in the patient characteristics were analyzed using the *t*-test for continuous variables and the chi-square test and the Fisher exact test for categorical variables. To assess the local efficacy, the complete resection rate for EMR and the complete response rate for PDT were evaluated and the confidence intervals (CI) were obtained using the Clopper-Pearson method, then compared using the Fisher exact test.

Two survival outcomes were measured: local recurrence-free survival (LRFS) and overall survival (OS). LRFS was defined as the period from the date of the initial SET until the first date of the detection of histologically confirmed local recurrence, and patients were censored at the time of the last endoscopy if they showed no local recurrence. OS was defined as the period from the date of the initial SET until the date of death from any cause, and patients were censored at the time of their last follow-up if they were alive. Survival curves were estimated using the Kaplan-Meier method and were compared using the log-rank test. Univariate and multivariate Cox proportional hazard models were used to examine the effect of clinical factors on LRFS and OS. All the statistical analyses were performed using IBM SPSS statistics 20 (IBM Japan Ltd., Tokyo, Japan). All the *P* values were reported as two-sided, with a significance level of 0.05.

## Results

### Patient characteristics

A total of 77 consecutive patients who had been treated with SET for local recurrence after CRT were enrolled in this study (Table 1). At the baseline before CRT, most of the patients had T1-T3 disease; however, seven patients with T4 disease were also included. Thirty patients (39 %) had lymph node metastases, and 7 (9.1 %) of them had non-regional lymph node metastases and were evaluated as having Stage IV disease at baseline. None of the patients in this study had distant organ metastasis prior to undergoing CRT. Most of the patients received a combination of cisplatin and fluoropyrimidine derivatives with concurrent radiotherapy of 50.4 Gy or 60 Gy.

**Table 1:** Baseline characteristics before chemoradiotherapy and salvage endoscopic therapy

Before chemoradiotherapy	Total (*n* = 77)		EMR (*n* = 39)		PDT (*n* = 38)		*P* value
Characteristics	Number	%	Number	%	Number	%	
Gender							1.000
Male	74	96.1	37	94.9	37	97.4	
Female	3	3.9	2	5.1	1	2.6	
Location							0.509
Upper	22	28.6	11	28.2	11	28.9	
Middle	41	53.2	19	48.7	22	57.9	
Lower	14	18.2	9	23.1	5	13.2	
cT factor							0.271
1	33	42.9	21	53.8	12	31.6	
2	10	13.0	4	10.3	6	15.8	
3	27	35.1	11	28.2	16	42.1	
4	7	9.1	3	7.7	4	10.5	
Lymph node metastasis							0.355
Absent	47	61.0	26	66.7	21	55.3	
Present	30	39.0	13	33.3	17	44.7	
cStage							0.394
I	27	35.1	17	43.6	10	26.3	
II	27	35.1	13	33.3	14	36.8	
III	16	20.8	6	15.4	10	26.3	
IV	7	9.1	3	7.7	4	10.5	
Before salvage endoscopic therapy	Total (*n* = 77)		EMR (*n* = 39)		PDT (*n* = 38)		*P* value
Characteristics	Number	%	Number	%	Number	%	
Median age (range)	65 (44–84)		63 (44–77)		67 (51–84)		0.038
Chemotherapy regimen							0.716
Cisplatin + fluoropyrimidine	71	92.2	36	92.3	35	92.1	
Nedaplatin + fluoropyrimidine	3	3.9	2	5.1	1	2.6	
Monotherapy	3	3.9	1	2.6	2	5.3	
Radiation dose							0.702
50.4	29	37.7	13	33.3	16	42.1	
60	43	55.8	23	59.0	20	52.6	
> 60	5	6.5	3	7.7	2	5.3	
Tumor depth evaluated with EUS							<0.001
uT1	67	87.0	39	100.0	28	73.7	
uT2	10	13.0	0	0.0	10	26.3	
Circumferential spread							0.002
< 1/4	45	58.4	30	76.9	15	39.5	
1/4 ≤, < 1/2	27	27.0	6	15.4	21	55.3	
1/2 ≤, < 3/4	4	4.0	2	5.1	2	5.3	
3/4 ≤	1	1.3	1	2.6	0	0.0	

*Abbreviations*: *EUS* endoscopic ultrasound, *EMR* endoscopic mucosal resection, *PDT* photodynamic therapy

At SET, the median age of the patients at the time of SET was 65 years, and the patients who underwent PDT were significantly older than those who underwent EMR (63.0 vs. 66.7, *P* = 0.038). Regarding the lesions before SET, ten patients (26.3 %) had a uT2 lesion in the PDT subgroup, and more patients had lesions with a larger circumference than those observed in the EMR subgroup.

### Local efficacy after SET

The local efficacy of SET is shown in Table 2. Among the patients who underwent EMR, a complete resection was achieved in 33 of the 39 patients, resulting in a complete resection rate of 84.6 % (95 % CI: 69.5–94.1). Among the six patients who did not achieve a complete resection after EMR, two patients had a positive horizontal margin, two patients had a positive vertical margin, and two patients had positive horizontal and vertical margins. Among the 13 patients who did not achieve a complete response after PDT, cancer cells were pathologically detected in the biopsy specimens soon after PDT in 12 of them, and one patient experienced a treatment-related death (described later). Although the complete resection rate for lesions occupying an esophageal circumference smaller than 1/4 was higher than those occupying an esophageal circumference of 1/4 or larger, the difference was not significant (90.0 % vs. 66.7 %, *P* = 0.089).

**Table 2:** Local efficacy rate of salvage endoscopic therapy

Endoscopic mucosal resection	Total	Number	Complete resection rate (%)	95 % CI	*P* value
All patients	39	33	84.6	69.5–94.1	
Circumference					0.089
< 1/4	30	27	90.0	73.5–97.9	
1/4 ≤	9	6	66.7	29.9–92.5	
Photodynamic therapy	Total	Number	Complete response rate (%)	95 % CI	*P* value
All patients	38	25	65.8	48.6–80.4	
uT factor					0.263
uT1	28	20	71.4	51.3–86.8	
uT2	10	5	50.0	18.7–81.3	
Circumferential spread					0.136
< 1/4	15	12	80.0	51.9–95.7	
1/4 ≤	23	13	56.5	30.6–73.2	

Abbreviations: *EMR* endoscopic mucosal resection, *PDT* photodynamic therapy, *CI* confidence interval

Among the patients who underwent PDT, a complete response was achieved in 25 of the 38 patients, resulting in a complete response rate of 65.8 % (95 % CI: 48.6–80.4). Although the complete response rate of patients with uT1 lesions was higher than that of patients with uT2 lesions, the difference was not significant (71.4 % vs. 50.0 %, *P* = 0.263). Similarly, although the complete response rate of lesions occupying an esophageal circumference smaller than 1/4 was higher than that of lesions occupying an esophageal circumference of 1/4 or larger, the difference was not significant (80.0 % vs. 56.5 %, *P* = 0.136).

### Clinical course after SET

**Fig. 1:**
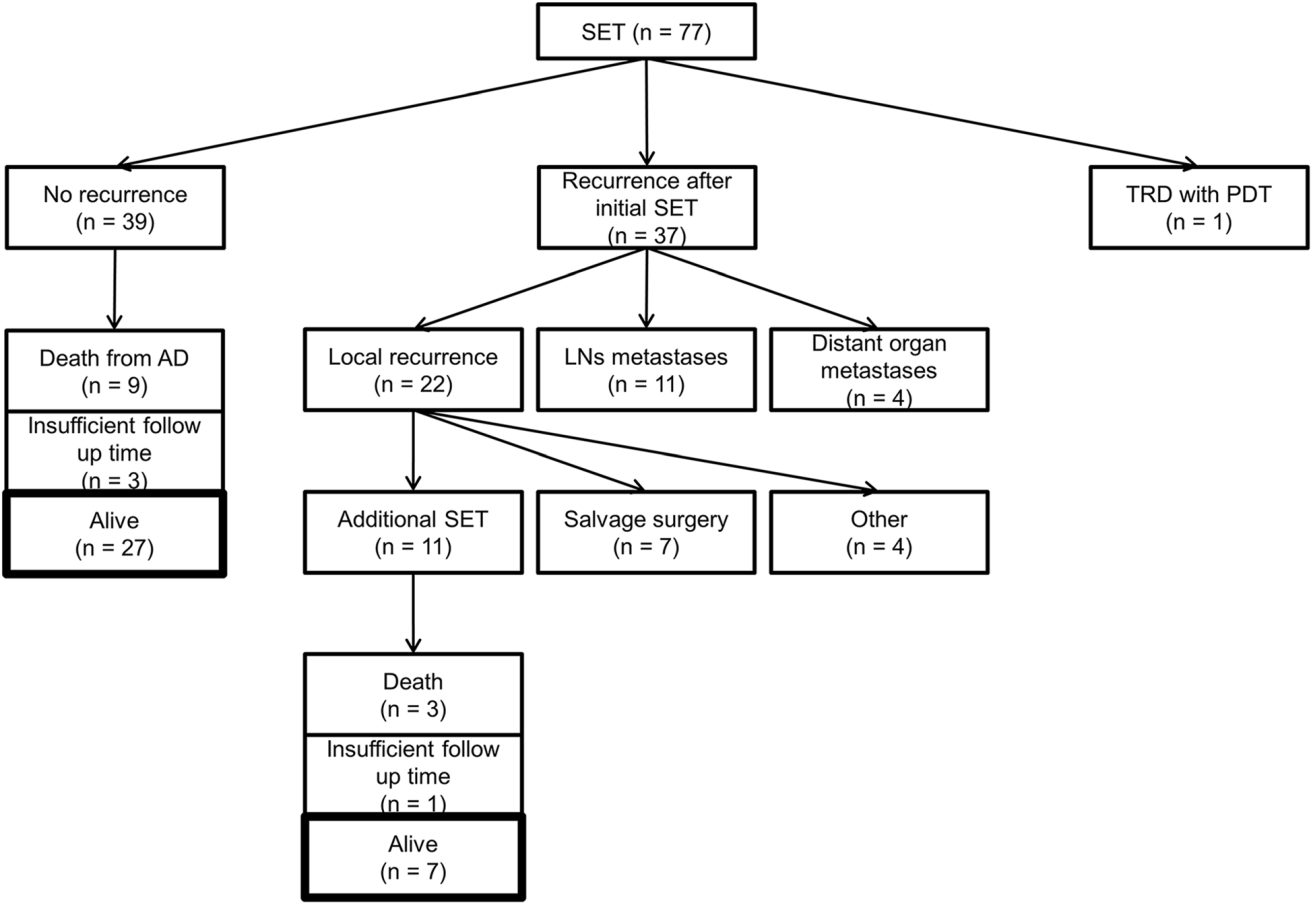
Clinical course up to 5 years after salvage endoscopic therapy

The median follow-up time of the censored patients was 6.7 years (range, 1.6–14.1 years) from the date of SET. The clinical course up to 5 years after initial SET is shown in Fig. 1. A total of 39 patients did not develop any recurrence, and 27 of them were alive at 5 years after the initial SET. A total of 37 patients developed recurrence at any site after SET, and local recurrence without any metastasis was detected in 22 of these patients: 7 after EMR, and 15 after PDT. Among them, 18 patients underwent additional salvage therapy with curative intent. Seven patients who consented to undergo surgery underwent salvage esophagectomy, and 11 patients who did not consent to undergo surgery underwent additional SET according to the same indication criteria as those used for the initial SET. In contrast, four patients who were assessed as not being capable of tolerating surgery and who did not fulfill the indication criteria for SET received chemotherapy with palliative intent or best-supportive care. Among 11 patients who underwent additional SET, seven were alive at 5 years after the initial SET. Overall, 34 of the 77 patients (44.2 %) recruited in this study survived for 5 years or longer after receiving only local treatment with SET. In addition to the seven patients with local recurrence, five patients with lymph node metastasis after SET underwent an esophagectomy with lymph node dissection and one patient with lung metastasis underwent a partial pneumonectomy. As a serious toxicity, one patient in this series who underwent PDT died as a result of a massive gastrointestinal hemorrhage caused by esophageal-aortic fistula formation that was possibly related to PDT.

In an analysis of the association between the resection margin and local recurrence in patients undergoing EMR, one of the six patients who had not achieved a complete resection and 11 of the 33 patients who had achieved a complete resection experienced a local recurrence.

### Survival outcomes after SET

**Fig. 2:**
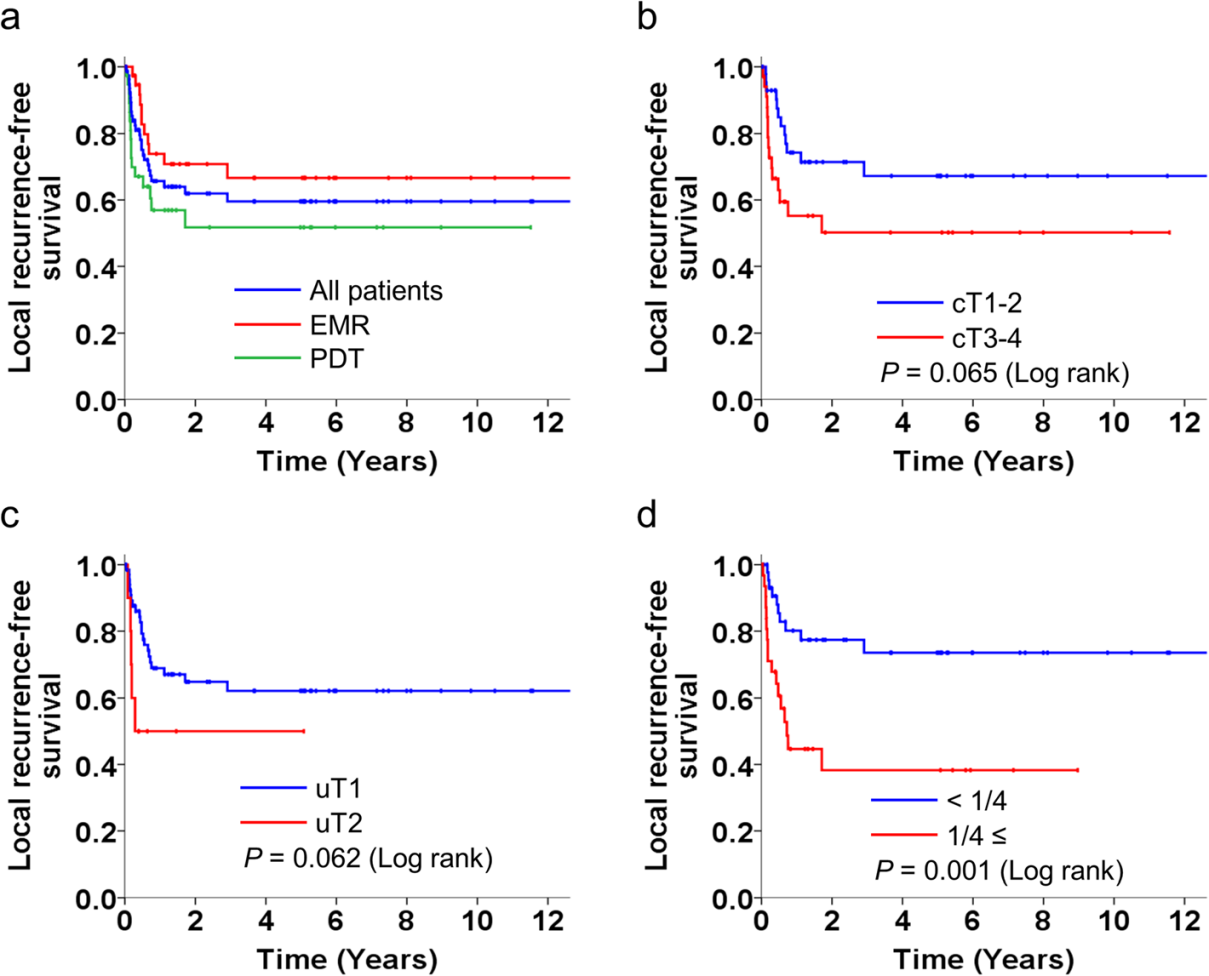
Local recurrence-free survival after salvage endoscopic therapy. **a** procedures. **b** cT factor before chemoradiotherapy. **c** uT factor before salvage endoscopic therapy. **d** circumferential spread before salvage endoscopic therapy

The 5-year LRFS rates of patients undergoing EMR, PDT, and all of the recruited patients were 66.7 % (95 % CI: 50.2–83.2), 51.7 % (95 % CI: 33.7–69.7), and 59.6 % (95 % CI: 47.4–71.8), respectively. The 5-year LRFS rate of patients with cT1-2 lesions was higher than that of patients with cT3-4 lesions, but the difference was not significant (67.2 % vs. 50.2 %, *P* = 0.065 [log-rank test]). The 5-year LRFS rate of patients with uT1 lesions was higher than that of patients with uT2 lesions, but the difference was not significant (62.2 % vs. 50.0 %, *P* = 0.062 [log-rank test]). The 5-year LRFS rate of patients with lesions occupying an esophageal circumference smaller than 1/4 was significantly higher than that of patients with lesions occupying an esophageal circumference of 1/4 or larger (73.5 % vs. 38.3 %, *P* = 0.001 [log-rank test]) (Fig. 2). In both univariate and multivariate Cox proportional hazard models, circumferential spread (1/4 or larger) was the only significant prognostic factor for local recurrence after SET (hazard ratio [HR]: 3.10, 95 % CI: 1.35–7.15, *P* = 0.008) (Table 3).

**Table 3:** Univariate and Multivariate Cox proportional hazard analyses

Characteristic	Number	Univariate			Multivariate		
		HR	95 % CI	*P* value	HR	95 % CI	*P* value
Local recurrence-free survival							
Location							
Upper	22	ref			ref		
Middle-Lower	55	0.95	0.41–2.16	0.894	1.13	0.49–2.60	0.777
cT factor							
1–2	43	ref			ref		
3–4	34	2.01	0.94–4.32	0.072	1.89	0.87–4.12	0.107
uT factor							
1	67	ref			ref		
2	10	2.55	0.95–6.86	0.065	1.21	0.42–3.46	0.725
Circumferential spread							
< 1/4	45	ref			ref		
1/4 ≤	32	3.38	1.54–7.41	0.002	3.10	1.35–7.15	0.008
Overall survival							
age							
≤ 65	39	ref			ref		
> 65	38	1.20	0.67–2.16	0.540	1.44	0.77–2.68	0.250
Location							
Upper	22	ref			ref		
Middle-Lower	55	0.91	0.48–1.74	0.779	1.14	0.57–2.28	0.721
cT factor							
1–2	43	ref			ref		
3–4	34	2.01	1.12–3.62	0.020	1.96	0.98–3.92	0.055
Lymph node metastasis							
Absent	47	ref			ref		
Present	30	1.59	0.88–2.88	0.123	1.14	0.58–2.27	0.701
uT factor							
1	67	ref			ref		
2	10	3.02	1.43–6.40	0.004	2.15	0.89–5.21	0.089
Circumferential spread							
< 1/4	45	ref			ref		
1/4 ≤	32	1.62	0.90–2.90	0.108	1.30	0.66–2.55	0.447

*Abbreviations*: *HR* hazard ratio, *CI* confidence interval

**Fig. 3:**
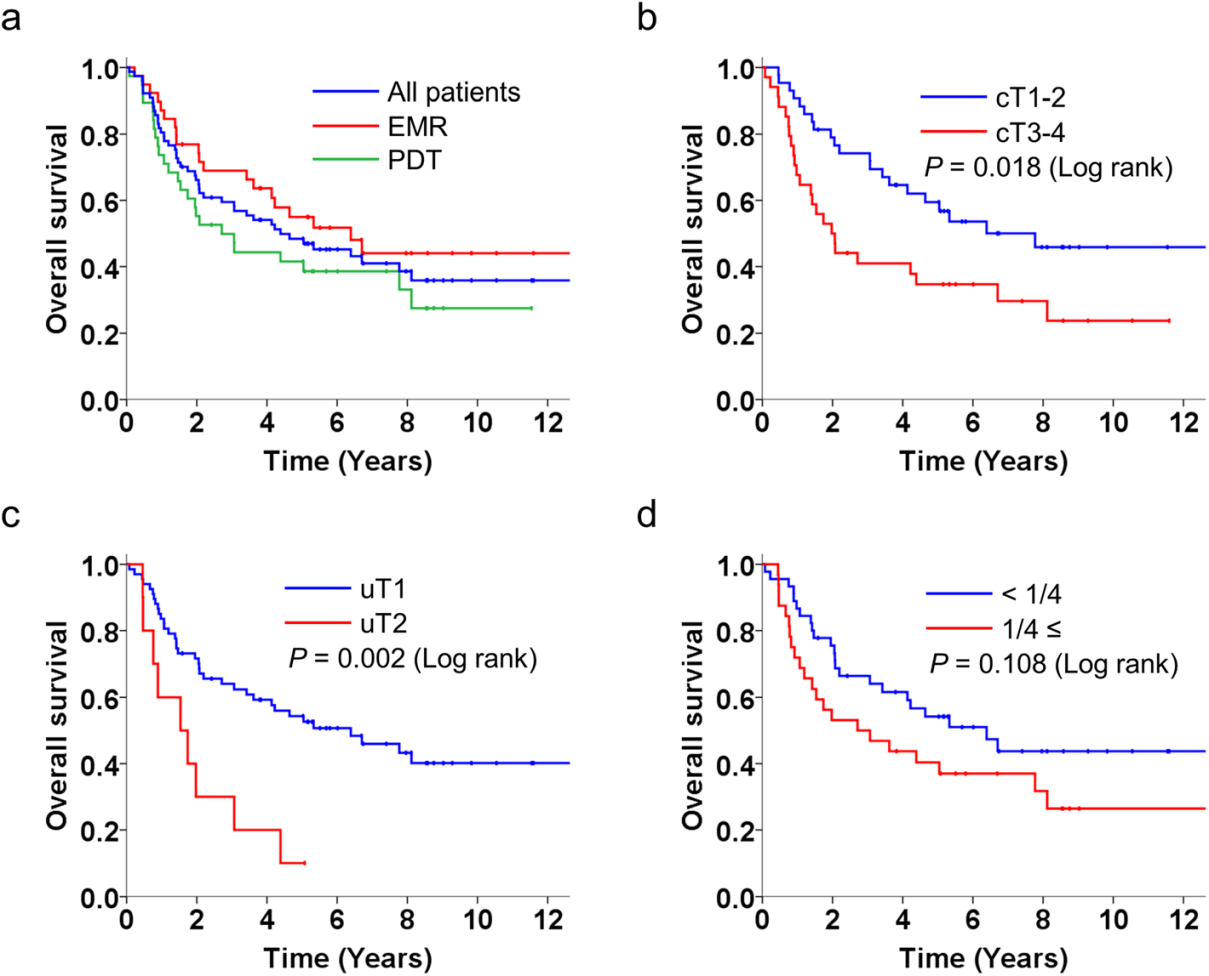
Overall survival after salvage endoscopic therapy. **a** procedures. **b** cT factor before chemoradiotherapy. **c** uT factor before salvage endoscopic therapy. **d** circumferential spread before salvage endoscopic therapy

The 5-year OS rates of patients undergoing EMR, PDT, and all of the recruited patients were 55.0 % (95 % CI: 38.9–71.1), 41.6 % (95 % CI: 25.7–57.5), and 48.4 % (95 % CI: 37.0–59.8), respectively. The 5-year OS rate of patients with cT1-2 lesions was significantly higher than that of patients with cT3-4 lesions (59.5 % vs. 34.7 %, *P* = 0.018 [log-rank test]). The 5-year OS rate of patients with uT1 lesions was significantly higher than that of patients with uT2 lesions (54.3 % vs. 10.0 %, *P* = 0.002 [log-rank test]). Though the 5-year OS rate of patients with lesions occupying an esophageal circumference smaller than 1/4 was higher than those with lesions occupying an esophageal circumference of 1/4 or larger, the difference was not significant (54.2 % vs. 40.4 %, *P* = 0.108 [log-rank test]) (Fig. 3). In a univariate Cox proportional hazard model, the cT factor (T3-4) and uT factor (uT2) were significant predictors of a poor OS. Although the cT factor demonstrated a tendency toward an association with a poor OS in a multivariate Cox proportional hazard model, the correlation was not statistically significant (HR: 1.96, 95 % CI: 0.98–3.92, *P* = 0.055) (Table 3).

### Discussion

In this study, we demonstrated that endoscopic interventions such as EMR and PDT are effective salvage treatment strategies for local recurrence after definitive CRT for ESCC, based on detailed data obtained over a long follow-up period. We also showed that a large circumferential spread before SET was a risk factor for local recurrence after SET, and lesions with an advanced T factor before CRT were likely to be associated with a shorter OS. We evaluated LRFS as the other survival endpoint in addition to OS in the present study, although to date local recurrence has not been confirmed as a surrogate marker for OS. However, local recurrence is a serious clinical event in patients with lymph node or distant metastasis, since quality of life, represented by oral intake, can be maintained if local control is achieved. The 5-year OS rates were relatively higher than those in our previous reports on both salvage EMR and PDT [12, 13]. These higher values were likely obtained because we recruited only patients with local recurrence who had previously achieved a complete response with definitive CRT and who were unlikely to experience recurrence in locoregional lymph nodes.

More than 40 % of the patients recruited in this study survived for 5 years or longer with only repeated SET, even if a local recurrence occurred after the initial SET. Several prospective studies of definitive chemoradiotherapy have demonstrated that failure in locoregional lymph nodes occurred much less frequently than local failure at the primary site, regardless of residue or recurrence [1, 18, 19]. In addition, Onozawa reported that patients with a complete response after CRT rarely experience recurrence in locoregional lymph nodes [11]. The results of the present study supported the importance of control for local recurrence after definitive CRT and showed that a certain proportion of patients who did not need salvage surgery were included in this population. SET might be a treatment option for local recurrence after definitive CRT, especially for patients who are unable to undergo surgery because of their poor medical condition.

When EMR was performed as an initial treatment for primary superficial ESCC, lesions with a large circumferential spread were associated with a positive resection margin and local recurrence [20, 21]. The elevated risk for local recurrence in patients undergoing SET for lesions with a large circumferential spread demonstrated in the present study was in agreement with the situation for initial EMR. However, local recurrence occurred relatively frequently, even in patients who achieved a complete resection with salvage EMR. In general, a local recurrent lesion arises from part of a primary lesion. Therefore, another recurrent lesion can arise later from the same primary lesion as a result of metachronous multifocal recurrence even if a complete resection of the initial recurrent lesion is achieved by salvage EMR. Careful endoscopic follow-up examinations are needed after initial SET, keeping the possible existence of occult recurrent lesions in mind. Regarding OS, an advanced cT factor before CRT and uT factor before SET seemed to be associated with a poor OS, though the correlations were not statistically significant. An advanced T factor before CRT has also been shown to be a poor prognostic factor in patients undergoing salvage surgery [7, 8, 22]. Considering that a significant proportion of patients who initially showed an advanced T factor before CRT obtained a long-term survival in the present study, such patients may be candidates for SET. In contrast, PDT was indicated for the treatment of uT1 and uT2 local failure lesions after CRT in clinical practice and in two separate prospective Japanese PDT studies [23, 24]. However, given the poor OS of the patients with uT2 lesions, the survival benefit of SET, namely, PDT is suggested to be small for those patients. The OS curve for a surgical cohort of patients with a local recurrent lesion of T1-2N0M0 disease after definitive CRT who underwent salvage surgery during the same time period is shown in Additional file 3: Figure S3. Although the 5-year OS rate was lower than that of the PDT cohort, we cannot make a simple comparison between the PDT cohort and the surgical cohort, given the small sample size and the underlying selection biases.

Although the long-term efficacy of SET in patients with local recurrence after definitive CRT has not been previously evaluated, an important limitation of the present study was that it was a single-institutional, retrospective study with a relatively small sample size. A prospective, multi-institutional observational study is needed to validate the presently reported results. Furthermore, the diagnostic accuracy of EUS is limited because of radiation-induced esophagitis and ulceration when performed soon after CRT [25]. In the present study, we only performed EUS for patients with a recurrent lesion after a complete response after CRT had been achieved and radiation-induced esophagitis or ulceration had disappeared. In addition, we previously demonstrated a high diagnostic accuracy of EUS for uT1 recurrent lesions after definitive CRT using a different cohort that underwent salvage EMR [26]. Although no diagnostic criteria for EUS in this setting have been established to date, the diagnosis of tumor depth using EUS may have been appropriate in the present study.

Recently, endoscopic submucosal dissection (ESD) has been applied as SET [27–29]. Not only may salvage ESD enable the performance of an en block resection, but it also may expand the indications for endoscopic resection for lesions that are not technically indicated for EMR because of their size or the presence of fibrosis. Although ESD as a treatment for primary superficial ESCC can enable an en block resection and reduce local recurrence [30–32], whether ESD as SET can demonstrate a similar efficacy has not been clarified. The accumulation of data on patients undergoing ESD as SET is also needed.

### Conclusions

In conclusion, we demonstrated the long-term efficacy of SET for local recurrence after definitive CRT for ESCC in an analysis of data obtained over a long follow-up period. SET can be a treatment option for definitive salvage treatment with appropriate patient selection through a comprehensive assessment of both the primary lesion before CRT and the recurrent lesion. Especially, careful endoscopic follow-up is needed in patients with large lesions before SET and before CRT, even if the local recurrent lesions are found to have been cured with SET.

## Additional files

Additional file 1: Figure S1. Representative endoscopic images of a patient undergoing salvage EMR. (TIF 2.19 mb) Additional file 2: Figure S2. Representative endoscopic emages of a patient undergoing salvage PDT. (TIF 2.71 mb) Additional file 3: Figure S3. Overall survival curve of the surgical cohort (*n* = 9). (TIF 147 kb)

## Abbreviations

CI: confidence intervals
CRT: chemoradiotherapy
CT: computed tomography
EMR: endoscopic mucosal resection
ESCC: esophageal squamous cell carcinoma
ESD: endoscopic submucosal dissection
EUS: endoscopic ultrasound
HR: hazard ratio
LRFS: local recurrence-free survival
OS: overall survival
PDT: photodynamic therapy
SET: salvage endoscopic therapy
