# Supplementary figures and images for: Local efficacy and survival outcome of salvage endoscopic therapy for local recurrent lesions after definitive chemoradiotherapy for esophageal cancer
Source: Radiat Oncol. 2016 Feb 27;11:31. doi: 10.1186/s13014-016-0604-z (PMC4769588; doi:10.1186/s13014-016-0604-z)

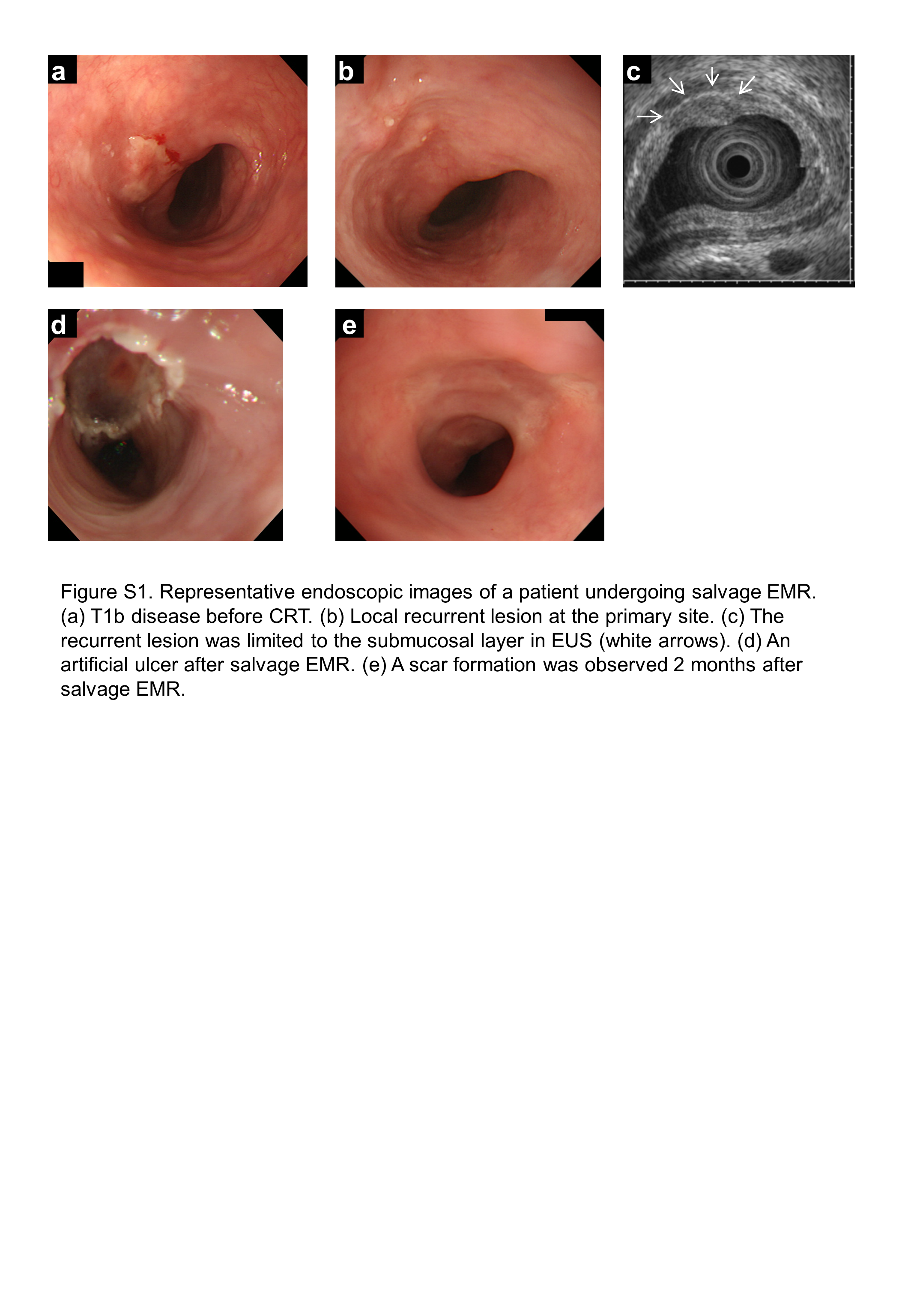

Supplement: Additional file 1: Figure S1. — Representative endoscopic images of a patient undergoing salvage EMR. (TIF 2.19 mb) [file 13014_2016_604_MOESM1_ESM.tif]

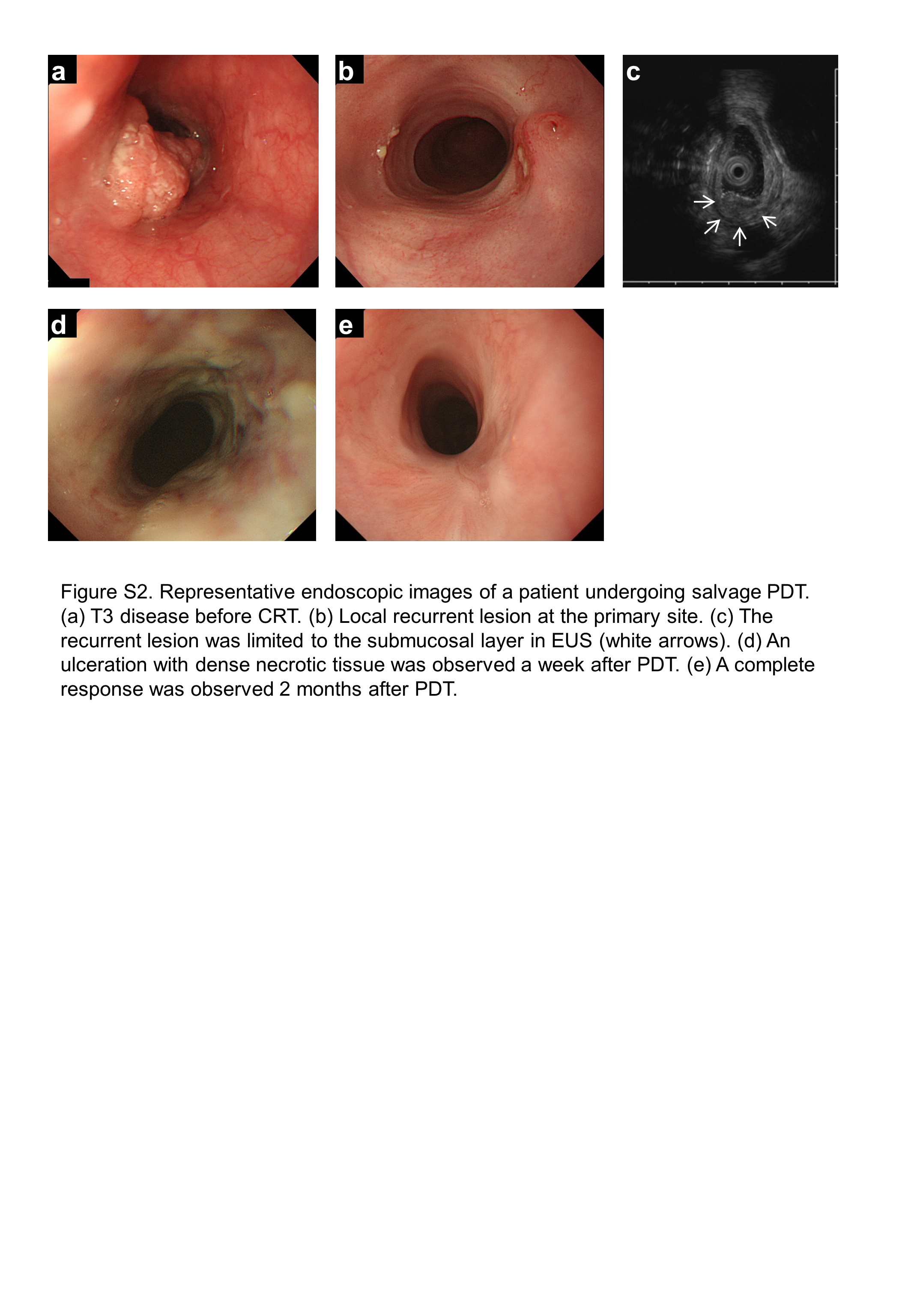

Supplement: Additional file 2: Figure S2. — Representative endoscopic emages of a patient undergoing salvage PDT. (TIF 2.71 mb) [file 13014_2016_604_MOESM2_ESM.tif]

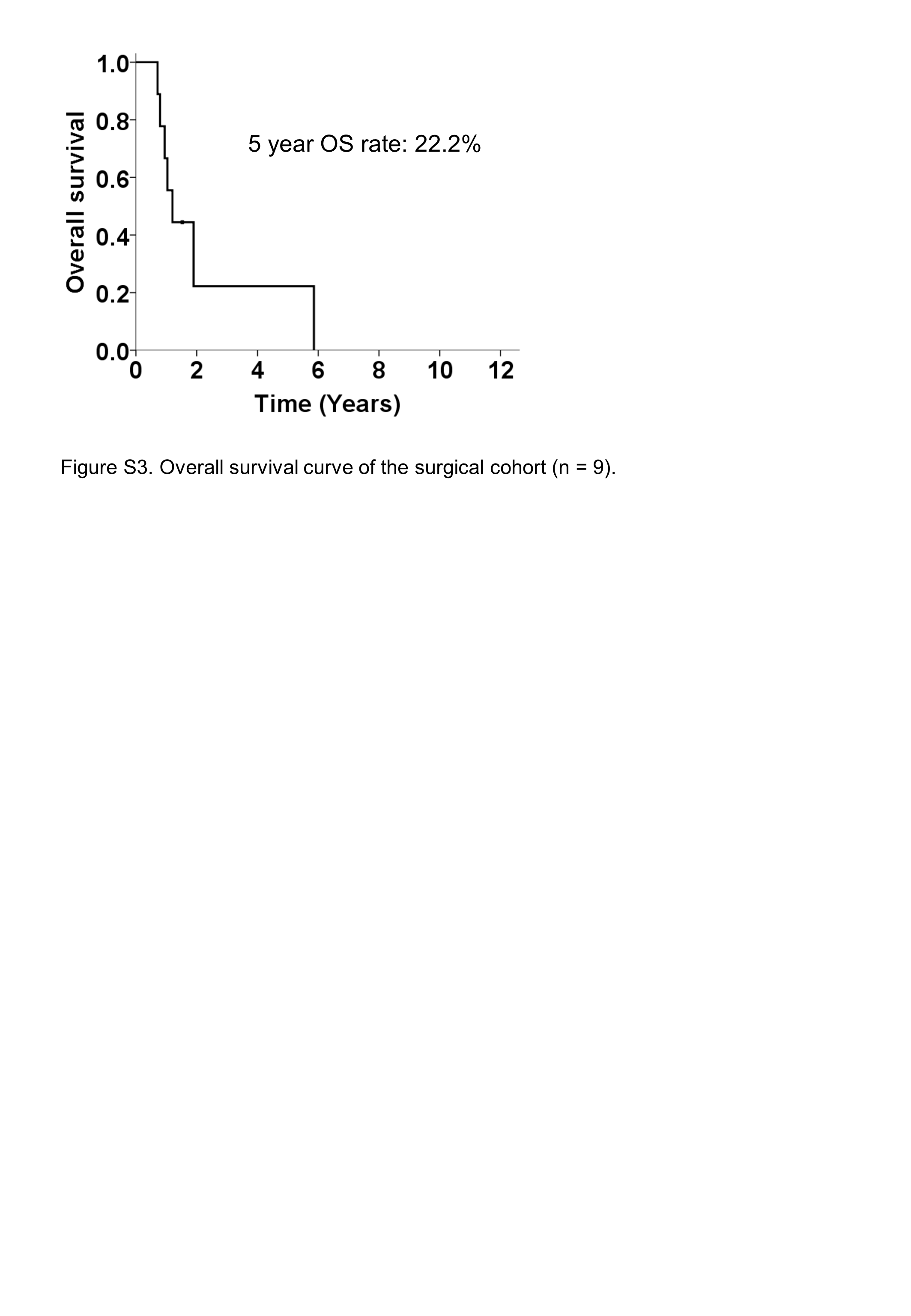

Supplement: Additional file 3: Figure S3. — Overall survival curve of the surgical cohort (n = 9). (TIF 147 kb) [file 13014_2016_604_MOESM3_ESM.tif]
